# Interactive visual analysis of a large ICU database: a novel approach to data analysis

**DOI:** 10.1186/cc9555

**Published:** 2011-03-11

**Authors:** H Gan, K Matkovic, A Ammer, W Purgathofer, D Bennett, M Terblanche

**Affiliations:** 1 Guy's & St Thomas' Hospital, London, UK; 2 VRVIS Research Centre, Vienna, Austria; 3 King's College London, London, UK

## Introduction

ICUs generate vast amounts of valuable data. The size and complexity of the data make analysis technically demanding and time-consuming. We used interactive visual analysis (IVA) to analyse a large ICU database using the association between sodium and mortality as a case study.

## Methods

We analysed routinely collected longitudinal clinical ICU data using ComVis^©^, an IVA tool developed for research in nonmedical fields. Coordinated multiple views enable the simultaneous visualisation of multiple variables of any data type (including time series). Individual variables and relationships between multiple variables are displayed in multiple linked views using user-selected box plots, histograms, scatter-plots, time series, parallel coordinates, and so forth. Visually selecting data by brushing with the cursor simultaneously highlights corresponding data in all other views. Multiple brushes are combined using Boolean logic, and the new selection is automatically updated across all views. We used IVA to analyse the univariate effect of sodium (Na) longitudinal trends (and rate of change) on mortality in 1,447 ICU patients. We defined high sodium as >150 mmol/l, low Na as <130 mmol/l, and a rapid rise and fall as a change >3 mmol/l/hour at any time. Trends of interest were identified using IVA while OR and *P *values were calculated using standard statistical techniques.

## Results

Overall ICU mortality was 22.5% (95% CI = 0.20.3 to 24.7%). Mean Na was 140 mmol/l (SD 4.3, within-patient minimum and maximum 123 and 166). Mortality was associated with: high Na versus Na <150 (28.6% vs. 20.9%, OR = 1.5, *P *= 0.004); rapid Na fall versus no rapid fall (27.6% vs. 17.7%, OR = 1.8, *P *< 0.001); and rapid Na rise versus no rapid rise (27.6% vs. 17.7%, OR = 1.8, *P *< 0.001). In contrast, low Na versus Na >130 (24.8% vs. 21.9%, OR = 1.2, *P *= 0.3), low Na with a rapid rise versus low Na with no rapid rise (26.3% vs. 20.7%, OR = 1.4, *P *= 0.3) and high Na with a rapid fall versus high Na with no rapid fall (30.6% vs. 24.2%, OR = 1.4, *P *= 0.3) were not associated with mortality.

## Conclusions

IVA facilitates a visual approach to data analysis that is both intuitive and efficient. This hypothesis can first be explored visually before further analysis using conventional statistical methods. Advanced statistical modeling can be used to confirm any potential hypothesis identified by visual analyses.

